# Leveraging WHO’s Global Benchmarking Tool to strengthen capacity in clinical trials oversight for public health emergencies: the GHPP VaccTrain model

**DOI:** 10.1186/s12992-022-00854-0

**Published:** 2022-06-20

**Authors:** Solomon Owusu Sekyere, Ivana Škrnjug-Yudov, Ulysse Ateba Ngoa, Marcela Juárez Hernández, Onome T. Abiri, James P. Komeh, Markieu Janneh Kaira, Essa Marenah, Juwe Darnuwele Kercula, Keturah Smith, Olga Rassokhina, Heidi Meyer, Christoph Conrad

**Affiliations:** 1 Paul-Ehrlich-Institut, Federal Institute for Vaccines and Biomedicines, Unit G3: International Coordination/Regulatory Services, Paul-Ehrlich Str. 51-59, 63225 Langen, Germany; 2 Paul-Ehrlich-Institut, Global Health Protection Programme (GHPP) RegTrain-VaccTrain, Langen, Germany; 3 Pharmacovigilance and Clinical Trials Department, Pharmacy Board of Sierra Leone, Central Medical Stores Compound, New England Ville, Freetown, Sierra Leone; 4 Medicines Control Agency, 54 Kairaba Avenue, K.S.M.D, Serrekunda, Gambia; 5 Medicines Information & Clinical Trials, Liberia Medicines & Health Products Regulatory Authority (LMHRA), Monrovia, Liberia; 6 Paul-Ehrlich-Institut, WHO Collaborating Centre for the Standardization and Evaluation of Vaccines & Global Health Protection Programme (GHPP) RegTrain-VaccTrain, Langen, Germany

**Keywords:** Emergency preparedness, Public health emergencies, Capacity strengthening, Clinical trials oversight, Evaluation, WHO GBT

## Abstract

**Background:**

A stable, well-functioning and integrated national medicines regulatory system is a core component of health systems resilient against infectious disease outbreaks. In many low- and middle-income countries, however, sizable gaps exist in the emergency preparedness framework of national regulatory authorities (NRAs). RegTrain-VaccTrain is a project of Germany Ministry of Health’s Global Health Protection Programme that contributes to global efforts aimed at strengthening such regulatory systems by providing technical support and advice to partner NRAs. In this study, we probed the outputs of our capacity-strengthening activities for clinical trials oversight (CTO) to take stock of progress made and examine remaining priorities in order to provide specialized technical assistance in addressing them to improve operational readiness for emergencies.

**Method:**

Data validated from NRA self-benchmarking results in 2017 and worksheet records of November 2021 were utilized to assess the emergency preparedness capacity for CTO in three VaccTrain partner NRAs (Liberia, Sierra Leone, The Gambia) before and after interventional capacity-strengthening partnership, using specific public health emergency-related (sub-)indicators of the WHO Global Benchmarking Tool.

**Results:**

A generally weak and vulnerable structural framework for CTO characterized the emergency preparedness capacity in all three partner NRAs at baseline, thus putting their operational readiness for public health emergencies at risk. VaccTrain’s collaborative work was successful at supporting individual NRAs to develop the full spectrum of operational structures (including (draft) regulations, guidelines, and standard operating procedures) required to improve regulatory preparedness. A gap in the formal approval and implementation of developed legal documents in two of three NRAs still remains. Notwithstanding, a robust emergency framework now exists and the NRAs stand better prepared to respond to (future) locally-concerning health emergencies, during which time clinical trials activity was observed to heighten.

**Conclusions:**

These results exemplify a north-south capacity-strengthening partnership model that effectively contributes in developing structures to enhance regulatory oversight and support expeditious product development in response to crises. They further underscore the equally critical role local/national processes play in facilitating the full implementation of developed structures.

**Supplementary Information:**

The online version contains supplementary material available at 10.1186/s12992-022-00854-0.

## Introduction

Regulation of medical products - as carried out by national regulatory authorities (NRAs) - is central to health systems and regarded as one of public health’s basic functions. It ensures that high quality, safe, efficacious, and cost-effective interventions like drugs, vaccines, and medical devices are accessible to all patients in times of need; a priority articulated in the United Nations Sustainable Development Goals [1]. The World Health Organization (WHO) duly acknowledges this and assigns regulatory system functions for medical products, vaccines and technologies as one of the six building blocks of developing a well-functioning health system [2].

Over the years, regulatory systems have endured public health emergency challenges, which often affect NRAs’ performance of essential functions. Clinical Trials Oversight (CTO) is one of such NRAs’ essential functions [3] which comes under serious strain in the wake of health emergencies [4–6]. During such crises, an enormous surge in global scientific research and development that culminates in rapid deployment of Clinical Trials (CTs) for diagnostics, therapeutics and preventative vaccines often ensue, posing an equally huge challenge on NRA capacities [6]. Under the circumstances, NRAs become particularly challenged to adapt to the use of specialized tools, processes and pathways to receive and expeditiously screen, evaluate, and authorize emergency-related CTs at greater speeds than regular routines. Unfortunately, however, capacity in many low- and middle-income countries (LMICs) for this pursuit is often limited and with considerable shortcomings [7, 8]. As a corollary of this weakness, the usually expected accelerated approval processes, which advances the availability and use of new medical products, vaccines and technologies at a faster pace is often hampered. From Influenza A H1N1, through Ebola, to SARS-CoV-2, experiences from public health emergencies have illustrated the urgent need to increase investments to strengthen CTO capacity in LMICs in a way to enhance NRA capacities to effectively prepare and respond to emergencies [8–11].

Strengthening regulatory capacities in LMICs to promote effective emergency responsiveness requires strong partnerships and regulatory collaborations. It is opined that functional emergency preparedness and responsiveness by NRAs in LMICs could be more effectively achieved if current efforts to strengthening them are supported and accelerated [8, 12]. Such capacity-strengthening partnerships and support systems, which the WHO asserts to be the most frequently mentioned priority in their country cooperation engagements [8], are vital in achieving impactful and sustainable public health outcomes and advancement of health and development [8, 13]. Compelling evidence suggests that NRAs’ robust regulatory capacity outcomes, which confer technical independence and strong mandate to authorize and supervise CTs, emanate from building expansive legal, organizational and structural architecture [14]. To help build strong CTO capacity on such pillars, the WHO’s Global Benchmarking Tool (GBT) provides a handy rubric to initially assess existing regulatory framework, formulate Institutional Development Plan (IDP) and target interventions to address the identified gaps [15]. Such was the strategy employed by the RegTrain-VaccTrain project of the German Federal Ministry’s Global Health Protection Programme (GHPP) at the Paul-Ehrlich-Institut in a structural capacity-strengthening intervention in three West African partner countries.

In this mini-evaluation exercise, we utilized emergency preparedness and capacity data collected with country-specific worksheets based on the WHO GBT to probe the outputs of our capacity strengthening activities for CTO in Liberia, Sierra Leone and The Gambia. The purpose was to take stock of the progress made with our intervention in improving regulatory preparedness for CTO in our three partner countries, examine the remaining priorities and provide specialized technical assistance, advice and capacity building opportunities to address them in order to improve responses to (future) public health emergencies.

## Results

### Weak emergency preparedness regulatory framework for clinical trials oversight at baseline

**Fig. 1:**
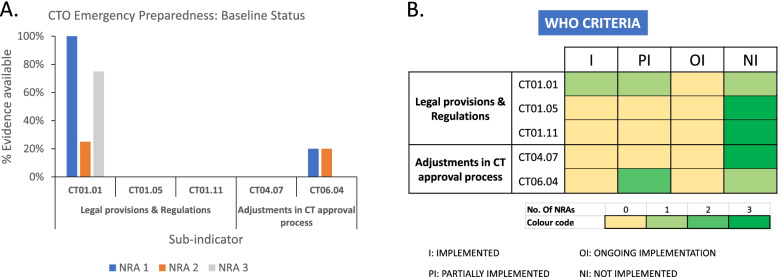
Baseline assessment of WHO Global Benchmarking Tool sub-indicators related to emergency preparedness for clinical trials oversight in VaccTrain’s partner NRAs. Validated self-benchmarking data collected in June – September, 2018 at the onset of our capacity-strengthening partnership was used to assess the availability and implementation status of public health emergency–related sub-indicators (i.e., CT01.01, CT01.05, CT01.11, CT04.07 and CT06.04) as per specific indications in the WHO GBT. **A** Status of partner NRAs’ emergency preparedness for CTO based on a scoring system that rated sub-indicators by percentage evidence available of individual “evidence to review” items. **B** Status of partner NRAs’ emergency preparedness for CTO based on WHO criteria which rated sub-indicators as not implemented (NI), ongoing implementation (OI), partially implemented (PI), or fully implemented (I)

WHO self-benchmarking is one of the early-stage operations of a stepwise strategy to an NRA’s capacity building initiative ideal to identify strengths and areas of weaknesses in a country’s regulatory system. Using worksheet data culled from self-benchmarking conducted in 2017 and validated by VaccTrain, we first assessed the emergency preparedness regulatory framework for CTO in our three partner countries prior to our capacity strengthening intervention. First, we employed our devised assessment scoring system (see Methods: section 7.4) to get a better understanding of the real structural shortcomings, which then needed to be fixed or improved (Fig. 1A). The ‘percentage evidence available’ for CT01.05, CT01.11 and CT04.07 were each found to be 0% for all NRAs, while CT06.04 had a paltry 20% evidence available by two NRAs and 0% by the other. CT01.01 was the only sub-indicator that had some appreciable implementation. This sub-indicator of the legal provisions and regulations that grant an NRA the fundamental legal mandate for CTO was found to be 100% implemented in one NRA, 75% in another, and 25% in the third (Fig. 1A). The latter NRA had no legal mandate to regulate CTs but had requisite guidelines for CTA submission, as per this sub-indicator. Clearly, the two NRAs with 100 and 75% implementation had laws with sections that specified the scope and extent of their mandate and stipulated that authorization from them is a legal requirement prior to initiating and conducting a clinical study. However, the accompanying requirement for guidelines that define the format and content of protocol, the procedure for submission, and the timeframe for review of application was missing in one NRA. Further analyzing the five sub-indicators using the WHO scoring system [15] (see Methods: section 7.4), an observation similar to the earlier findings were made. Aside CT01.01 which was fully implemented in one NRA, all the other sub-indicators were either not implemented (*n* = 11), or partially implemented (*n* = 3) (Fig. 1B). Altogether, this baseline assessment revealed a generally fragile emergency preparedness regulatory framework for CTO in our partner countries and further identified specific areas with significant gaps for capacity strengthening.

### Revamped clinical trials oversight’s operational structures for emergency preparedness upon VaccTrain’s capacity strengthening intervention

**Fig. 2:**
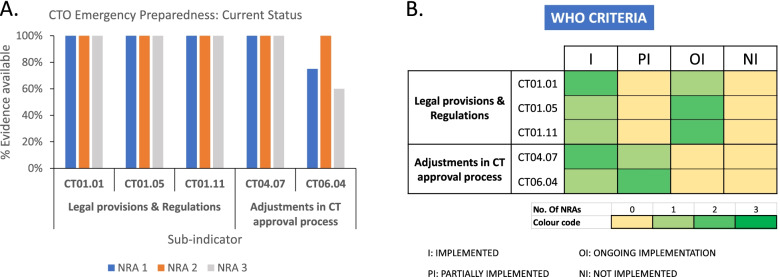
Effect of regulatory capacity strengthening on improving operational structures for clinical trials oversight in preparedness towards public health emergencies. The availability of structures and implementation status of WHO GBT sub-indicators related to CTO during public health emergencies were assessed in November 2021. **A** Current status of partner NRAs’ operational readiness for public health emergencies using the percentage evidence available scores of individual “evidence to review” items. **B** Current status of partner NRAs’ operational readiness for public health emergencies using the WHO criteria

Guided by the aforedescribed findings at baseline, we next assessed the availability and implementation status of public health emergency–related sub-indicators after some 2.5 years of our interventional capacity-strengthening partnership. This evaluation reveals that unlike the status at baseline, none of the sub-indicators analyzed at this time-point had a 0% implementation status. From our ‘percentage evidence available’ assessment using the scoring for individual ‘evidence to review’ components of each sub-indicator, we observed that CT06.04 was the only sub-indicator with outstanding gaps as all the others achieved a 100% scoring using this evaluation scheme (Fig. 2A). Nevertheless, this evaluation was only based on a crude assessment, which merely took stock of the availability of individual ‘evidence to review’ operational structures, and not necessarily their implementation. Using the WHO criteria of rating which also factored the implementation of the individual component items over time, we observed that a considerable number of sub-indicators were either partially implemented or under ongoing implementation although none of them remained ‘not implemented’. Specifically, *n* = 3 and *n* = 5 sub-indicators (out of the total 15 for the three NRAs together) displayed either partial or ongoing implementation respectively, while *n* = 7 was fully implemented (Fig. 2B). Notably, all sub-indicators that were rated as ‘ongoing implementation’ were those that were legal provisions-related (i.e. CT01.01, CT01.05, CT01.11) whereas the ‘partially implemented’ ones related to those that assessed adjustments to the routine CT authorization process (i.e. CT04.07, CT06.04) (Fig. 2B). Further to the observation of an improved operational readiness to public health emergencies, we sought to probe its relevance and likelihood of practical application in the event of emergencies of local concern in our partner countries. To do this, we utilized data from the registry and results databases *clinicaltrials.gov* and *pactr.samrc.ac.za* to assess the frequency dynamics of CTs related to major disease outbreaks such as Influenza A H1N1, Ebola and SARS CoV-2 in the regulatory jurisdictions of the individual NRAs. While there were no records of any Influenza H1N1-related CTs conducted during the pandemic years of 2009–2010, the era of Ebola epidemic in West Africa witnessed considerable number of trials in our partner countries (*n* = 23), many of which were interventional (*n* = 18) (Table 1). For the ongoing covid-19 pandemic, there have been a combined total of *n* = 5 CTs underway in the partner countries, 1 of which is interventional.

**Table 1:** Number of emergency related clinical trials on Influenza A H1N1, Ebola and SARS CoV-2 conducted in VaccTrain’s partner countries under study

		Number of emergency-related clinical trials conducted in respective countries, n (Interventional; Observational)		
Public Health Emergency	Period	NRA 1	NRA 2	NRA 3
The Influenza A (H1N1) pandemic	2009–2010	0 (0;0) ^c^	0 (0;0)	0 (0;0)^a^
The Ebola epidermic in West Africa	2013–2016	9 (7;2)	14 (11;3)	0 (0;0)^b^
The ongoing Covid-19 pandemic	2019 – ongoing	1 (0;1)	1 (0; 1)	3 (1;2)

Source: www.clinicaltrials.gov; www.pactr.samrc.ac.za

^a^ Agency not established yet; ^b^ Agency established a year before epidemic declared ended; ^c^Agency established in last year of pandemic

Overall, these data underscores the contributory role of VaccTrain’s partnership in resourcing LMIC partner NRAs with emergency preparedness operational tools for CTO in order to help them respond to disease outbreaks and public health emergencies in an effective manner. It further showcases the potential application of the tools in emergency situations to help the NRAs contribute to speeding up the product development process with regulatory agility.

### Significance of formal approval and implementation of developed structures in maximizing capacity strengthening gains

**Fig. 3:**
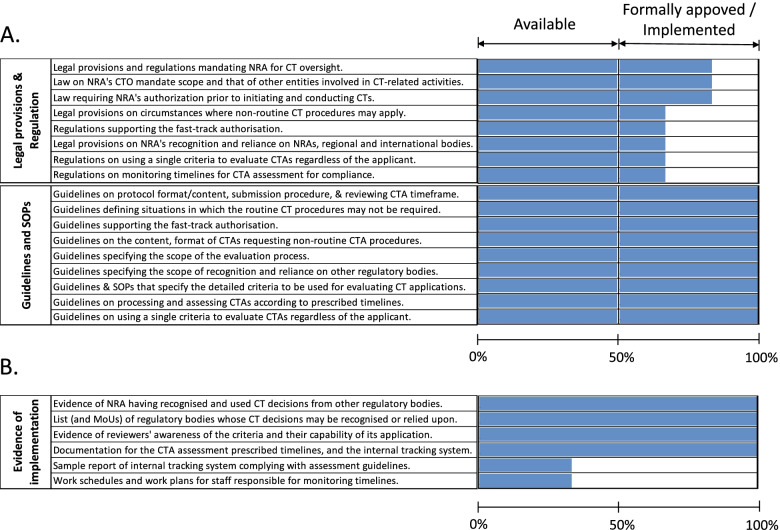
Formal approval and practical implementation status affecting capacity-strengthening achievements for clinical trials oversight-related emergency preparedness. The formal approval of CT legal provisions and regulations as well as their evidence of implementation and that of applicable guidelines and SOPs were investigated. Each ‘evidence to review’ item was scored (i.e., evidence provided = 1; no evidence provided = 0) and the total tally for the three NRAs unified was calculated as percentage evidence available. **A** The current status of all individual legal provisions and regulations (top panel), guidelines and SOPs (lower panel) of the emergency-related sub-indicators analyzed. **B** Evidence of implementation status of the emergency-related five sub-indicators analyzed

To provide more clarity on the developed regulatory documents and their effect on the achievement of full implementation status of the specific sub-indicators based on the WHO scoring, we acknowledged the different approval processes for the different regulatory documents and categorized them accordingly for further analysis. Essentially, we pooled all the ‘evidence to review’ items of the five emergency-related sub-indicators together and re-categorized them into three groups including (i) legal provisions and regulations (ii) guidelines and Standard Operating Procedures (SOPs) and (iii) evidence of implementation. We then assessed the legal provisions and regulations not only based on the availability of their ‘evidence to review’ items but also by their formal approval status (Fig. 3A). In doing so, we scored each ‘evidence to review’ item as described in the methods section (i.e., evidence provided = 1; no evidence provided = 0) and calculated the ‘percentage evidence available’ from the total tally of the three NRAs unified. Generally, three major observations were apparent from this analysis. First was the observation that all three partner NRAs currently possess the full gamut of key regulatory structures (including regulations, guidelines and SOPs) required to prepare and mount an effective CTO response to public health emergencies (Fig. 3A). The second was that although all three partner NRAs can now boast of the full complement of emergency preparedness regulatory structures, their formal approval status and current use vary between NRAs. As at the time of this evaluation, only one of three NRAs had managed to obtain the formal approval of the developed documents while their approval in the other two countries lingered. Evidence for the application and use of developed structures was fairly available (Fig. 3B). There was, however, a noteworthy indication that implementation deficiencies in the use of the internal tracking tool for CTA assessment and limitations in the work schedules and work plans for staff responsible for monitoring timelines remained as outstanding gaps. In summary, this data demonstrated the essence of complementing structural capacity strengthening efforts with support for the formal approval and implementation processes to reap the full gains of such co-operations aimed at improving regulatory preparedness for public health emergencies.

## Discussion

CT regulation in public health emergency situations is particularly challenging for NRAs with regards to the incredible pressure it comes with expediting assessments whiles ensuring safety in a time of tremendous uncertainty. To help NRAs (especially those from LMICs) to adequately prepare for such situations, investments in strengthening CTO capacities that will enable the effective absorption of this shock and enhance operational readiness to respond to emergencies is critical. This mini-evaluation here conducted in three of our partner countries, some 4 years after our capacity-strengthening partnership, reveals findings relevant to global collaborations for such endeavors. Our results show that the emergency preparedness and response framework for CTO in the three partner NRAs was especially weak and vulnerable at baseline, thus putting the operational readiness for disease outbreaks and public health emergencies at considerable risk. It further demonstrated that VaccTrain’s capacity-strengthening partnership contributed to the development of relevant technical structures (including (draft) regulations, guidelines and SOPs) as presently available for public health emergency response in all three partner NRAs. Finally, it revealed the significant role formal approval of developed regulatory structures and their subsequent implementation play in fully achieving defined outcomes of capacity-strengthening interventions and improving an NRA’s maturity level as per WHO’s ratings.

The process for strengthening any core component of a health system must follow an iterative cycle [8]. From the WHO 5-step capacity building model, this cycle starts with an initial gap analysis by a self-benchmarking exercise using the WHO GBT. This globally standardized instrument is known for its well-tested methodology for objectively benchmarking regulatory systems, generating IDP for addressing gaps through various technical support systems, and for monitoring progress, evaluating, and taking corrective actions. The VaccTrain aligns its capacity strengthening strategy with WHO in this regard and leverages this tool to direct our input at supporting the revamping of specific areas that need improvement.

The lack of capacity, resources and an effective preparedness and response plan in place for disease outbreaks came to the fore after the Ebola crises in West Africa highlighted the limitations in the world’s preparedness for public health emergencies [8]. Quite recently, data from WHO benchmarking results showed the potential fragility of many member states (especially LMICs) to public health threats due to limited regulatory preparedness [7]. Particularly for CTO, a full implementation of the sub-indicator for reliance on CT decisions of others was 23%; the least percentage implementation of the regulatory functions assessed for the 84 countries included in the study [7]. Our baseline data indicative of a fragile CT regulatory structure for timely approval of CTAs in our partner NRAs (all LMICs) at the inception of our capacity strengthening partnership, was therefore in consonance with these findings. A weak public health emergency-related regulatory ecosystem for CTO in LMICs means that during emergency situations clinical trial participants in these countries may be vulnerable to abuse due to laxities in regulatory enforcement and oversight. As the integrity of data generated from such studies may also be compromised, there may be an increased potential of consequential harm to individuals globally that may use medical products approved through such systems [16]. Without an effective and fully operational regulatory system, an LMIC becomes an unattractive destination for well-meaning product developers to conduct clinical trials. This impedes the advancement of the current trend of globalization of clinical research and hinders entire populations (locally or globally) from the rapid access to new medicines, vaccines and other health products that are often direly needed during public health emergencies and as part of sustainable development globally. Further, if clinical research are not conducted in LMIC settings as a result of weak infrastructure, they (i.e. the LMICs) miss out on certain indirect benefits that are sometimes associated with international clinical trials initiatives. Such indirect benefits could be capacity strengthening components in the area of infrastructure, education or training, which may altogether support continuity in research or improve regular medical care upon completion of the initial studies [17]. The limited number of countries involved in this study notwithstanding, its extension on the number of public health emergency-related sub-indicators for CTO as analyzed previously [7] is a valuable addition that strengthens the current body of knowledge on the status of emergency preparedness, particularly in LMICs. Aside the limitations in the reliance model on CT decisions of other competent NRAs during emergencies, this data further highlighted other CT regulations-related gaps as well as cracks in the adjusted procedures for expeditiously screening, evaluating and authorizing emergency-related CTs.

Since the Ebola outbreak in 2014, regulatory capacity strengthening in LMICs has attracted much attention as a global priority [8, 18]. The idea of the need for global collaborations to foster the development agenda is being further promoted [7, 12]. In our bid to support partner NRAs build emergency measures to improve access to essential health products and technologies for response and mitigation, the VaccTrain employs a strategy that thrives on the development and implementation of regulatory structures/documents as its centerpiece. For CTO, not only did we provide technical assistance in addressing deficiencies in the legal backbone for regulatory oversight, but we also lent support in addressing various gaps in CT guidelines, templates (i.e. forms) and internal SOPs as per the precepts of the GBT. This evaluation revealed that our collaborative work yielded tremendous success in developing all the CT regulations, guidelines and SOPs related to emergency preparedness and response. Being a technical partner of the African Vaccine Regulatory Forum (AVAREF), VaccTrain ensured that all our partner NRAs aligned their CTO emergency pathways with AVAREF’s and incorporated all regulatory documents developed by the forum. For emergency preparedness, these documents included the AVAREF Strategy and Guidance for Emergency Preparedness and Guideline for Joint and Assisted Reviews of Clinical Trial Applications (CTAs). Other documents for routine CTO such as those for validation and screening (i.e., AVAREF CT application form and Checklist), Inspection (AVAREF Good Clinical Practice Inspection Guide and Checklist) and for CTA assessment (i.e., Quality, Nonclinical, and Clinical, Statistical) were also adopted. By this, we contribute to establishing a harmonized regulatory framework on the continent to deal with health emergencies whiles avoiding duplication of efforts.

It is generally known that the various levels of capacity needs are interdependent. Aside the direct impact of revamped operational structures for CTO, improvements in human resource capacity that facilitates the practical utilization of the developed documents contribute to improving regulatory performance [19–23]. Quite recently, the central role of quality technical structures in translating training acquired knowledge into measurable CTO outcomes was reported [24]. This observation, together with our current findings, shows that the availability of the required technical structures and workforce with appreciable capacity work in concert to help implement capacity strengthening outcomes and maximize regulatory performance. Within the study period of this report, CTO staff from the various NRAs received various VaccTrain-sponsored trainings including the flagship FDA Ghana regional center of regulatory excellence (RCORE) CT training fellowship. It is therefore plausible that the staff training component of our multi-pronged capacity strengthening approach contributed to the enhanced CTO emergency preparedness infrastructure relative to that at baseline. In this vein, it would be worthwhile for future studies to explore other factors that could affect the change as observed in the before-after assessment.

Quite recently, a database surveillance study of vaccine-related CTs in Africa revealed Ebola virus disease as one of the four most studied diseases on the continent [25]. This observation, in part, clearly affirmed the global trend of migration of CTs towards disease endemic regions as had been previously reported [26–29]. Given that all three VaccTrain partner NRAs included in this study are from West Africa and two were heavily hit by the Ebola epidemic, our observation of elevated CT activity for Ebola virus disease between 2014 and 2016 (Table 1) was of no surprise. It however brought to the fore the dire relevance the current improved operational readiness for CTO could be put to use in mitigating regulatory challenges that have characterized the conduct of clinical trials in years past during public health emergencies [30, 31].

From this evaluation, it also became apparent that successful development of regulatory documents and their application/implementation are two distinct ventures that must be tackled at individual levels. VaccTrain had this foreknowledge and allotted a 2 year period (from 2019 to 2021) as the implementation phase of our CTO project’s structural development output. As the implementation phase coincided with the covid-19 pandemic, we took the opportunity to provide further assistance in the form of an online simulation exercise for emergency responsiveness in CTO, conducted an on-line training in advanced regulatory aspects of CTO and further sponsored the on-site training of partner NRA staff at FDA Ghana to enhance CTO regulatory capacity. Importantly, and relative to the structural framework for CTO, we supported stakeholder meetings organized by some of the NRAs to discuss draft CT guidelines before their full rollout. These processes are particularly important in the sense that procedures that waive certain requirements of routine conditions may be misunderstood as skimping measures that may compromise on quality. If such delicate matters are not adequately discussed with all stakeholders, their subsequent application may decrease public trust and confidence in the regulatory system and lead to the politicization of regulatory decisions. More also, such stakeholder discussions help to inform or update product developers of the various regulatory pathways they can seek to activate for the approval of their clinical research. Aside this endeavor, we also encouraged partner NRAs to strive locally for the formal approval of developed CT regulations (as this was outside VaccTrain’s scope) during the implementation phase. Up until now, only one of the three partner NRAs has been successful at securing the full Ministerial approval of their CT regulations, with that of the other two NRAs at various stages of the approval process. This lack of formal approval of CT applicable regulations accounted for the ‘ongoing implementation’ (and not ‘fully implemented’) status as observed for two of our partner NRAs in relation to the legal provisions-related CT sub-indicators (CT01.01, CT01.05 and CT01.11). The tardiness in getting the CT legal documents (as developed by our partnership) formally approved and implemented was an obvious concern. On the one hand, this inertia could be envisaged to be a result of the sheer bureaucratic tendencies that are often rife in many political quarters, especially regarding the adoption of legal documents. On the other hand the lack of political will, and sometimes a disconnect between the Ministry of Health and the NRAs can breed passivity and lethargy, which may delay the approval process, as that observed in this study. Also of importance is the way some governments view clinical research and the premium they put on it as an enabler for health improvement. The WHO GCP guidelines highlight the role of governments as one to provide the legal framework for clinical trials [32]. However, while some LMIC governments may consider clinical research as vital in achieving development goals and meeting the objectives of universal health coverage, others may have quite neutral (or sometimes hostile) stance towards it, regarding it as unnecessary, interfering or potentially problem-creating rather than problem-solving [17]. These differences, together with those in the formal approval pathways in the different regulatory jurisdictions, means that some get approved faster while others may require extra support in advocacy to facilitate approval and enforcement. This data further confirmed earlier findings that building capacity to conduct effective CT (or its oversight in this case) and achieving its full benefits is a process that grinds slowly and requires a long-term perspective [12]. Capacity building projects that seek to build an effective, impactful and sustainable CTO infrastructure in partner NRAs should therefore be guided by this fact and factor it into their planning.

This study has considerable limitations that have to be highlighted. First, it reported the emergency preparedness framework for only one regulatory function (i.e. CTO) although others such as Registration and Marketing Authorization, Pharmacovigilance and Regulatory Inspection are equally important. Second, the strict and technical nature of the criteria employed for this evaluation narrowed the scope of sub-indicators and excluded those related to the oversight of approved and ongoing CTs. It also excluded other important sub-indicators such as those that promote effective communication, accountability, and transparency as well as those that relate to staff training and performance. Third, this evaluation was short at providing insights at the outcomes level of the capacity strengthening partnership in fostering an effective approval process of CTAs during public health emergencies using the tools developed at the output level. As a next step, we aim to conduct a comprehensive evaluation, which will address the shortcomings of these findings and provide a better insight into the resilience of the whole CTO regulatory establishment in our partner NRAs, and how this helps in facilitating an effective and efficient authorization and oversight of CTs. The aforementioned limitations notwithstanding, the exhaustive nature of analysis, which encompassed all the public health emergency-related indicators for clinical trials oversight in the WHO GBT, was a major strength as it extended the scope of previous studies [7]. Further, the systematized results-oriented capacity-strengthening strategy that leveraged an established WHO model to achieve success in developing the full spectrum of emergency preparedness regulatory tools is another strength worth highlighting.

## Conclusion

In summary, this evaluation to assess the regulatory preparedness framework for public health emergencies in three VaccTrain partner NRAs before and after interventional capacity-strengthening partnership revealed a generally weak structural establishment at baseline. Using a worksheet approach to systematically address priority areas of improvement, our collaborative work was successful at developing the spectrum of operational structures required to improve regulatory preparedness for public health emergencies. The data also demonstrated that ample time is needed for formal approval and implementation of the structures in order to record any benefits at the outcome level. These findings give valuable insight into a results-oriented capacity-building model effective at developing regulatory tools to enhance operational readiness for public health emergencies.

## Methods

### GHPP RegTrain-VaccTrain regulatory capacity strengthening strategy: VaccTrain I

#### The project

RegTrain-VaccTrain is a project of the GHPP; a programme established by the German Federal Ministry of Health (Bundesministerium für Gesundheit, BMG) to assist partner countries and the WHO in epidermic prevention measures. In its pilot phase, so named *VaccTrain I*, the project focused on capacity development activities in the area of CTO in selected partner countries. The approach adopted included:

(i) providing technical assistance in support of the development and implementation of regulatory structures and processes as defined in NRA internal documents and legal framework
(ii) enhancing the human resource capacity for CTO through African Union Development Agency-New Partnership for Africa’s Development (AUDA-NEPAD)‘s-instituted Regional Center Of Regulatory Excellence training framework like that by FDA Ghana
(iii) supporting regional and pan-African harmonization initiatives through technical support to AVAREF.

In August 2017, the VaccTrain I project launched a call for proposals and selected the Economic Community of West African States (ECOWAS) countries Ghana, Liberia, Sierra Leone and The Gambia as partners, after the countries had expressed the desire to strengthen their capacity for CTO and affirmed their commitment to work in partnership with each other. The project kicked off in June 2018.

#### Description of the worksheet approach

An important aspect of this arm of the project was the assessment and analysis of the CTO structural architecture in the individual partner countries prior to our capacity strengthening intervention. As all our three partner countries had undertaken a WHO self-benchmarking in 2017, we capitalized on the results and the IDP generated to develop working documents so called NRA worksheets. These specialized NRA worksheets were designed with specific sections clearly defining and documenting details including (i) the partnership objective, (ii) score from last NRA assessment, (iii) description of review points (evidence provided), (iv) gaps identified, (v) NRA priority areas, (vi) expected deliverables and (vii) the methodology to employ in addressing gaps. This information was documented for each of the 30 sub-indicators of the GBT for CTO thus offering us a deeper understanding of the regulatory needs of the individual partner NRAs and allowing us to customize support for addressing lapses identified in the IDP. The worksheets were also helpful to document granularities of our operations, generate situational reports as well as monitor and evaluate progress against the 2017 self-assessment as a benchmark.

#### Technical support in development of regulatory structures for CTO

As a starting point, VaccTrain worked with individual partner countries to validate findings reported in the WHO self-benchmarking through initial country visits. During these country visits, we collaborated with partner agencies to identify gaps and deficiencies in the CTO operational framework, prioritized areas of improvement, determined expected deliverables (or outputs), agreed on the preferred methodology and set to addressing them via remote and on-site engagements. We began our support for the development of missing regulatory documents by diving headfirst assisting the drafting of CT regulations, guidelines and applicable templates, as well as SOPs. In all cases, we ensured that developed regulatory structures aligned with pan African (e.g. AVAREF requirements) and international standards and integrated best practices within the framework of good regulatory practice. To help facilitate implementation, VaccTrain sponsored a two-week on-site placement of selected partner NRA staff to FDA Ghana (an advanced regulatory authority in the ECOWAS region) where they understudied the practical application of the developed tools. We also organized various on-line workshops in advanced regulatory aspects of CTO and sponsored the training of partner NRA staff in FDA Ghana’s RCORE CT training fellowship to enhance human resource capacity in CT scientific assessments.

### The WHO global benchmarking tool for evaluating national regulatory systems

The Global Benchmarking Tool (GBT) is a globally standardized assessment tool developed by the WHO to serve as the primary means of objectively evaluating regulatory systems, as per provisions of the World Health Assembly Resolution 67.20 on Regulatory System Strengthening for medical products. It was designed by unifying previous WHO, Pan-American Health Organization (PAHO) and other non-UN agency parallel evaluation tools ( [15]. The tool is useful in its application to evaluating the broadscale regulatory framework and its modular regulatory functions (e.g. clinical trial oversight as in this study). For the overarching national regulatory system, as well as individual regulatory functions, there are specific (i) indicators, (ii) sub-indicators and (iii) fact sheets that help to measure an NRA’s regulatory capacity whiles ensuring a consistent evaluation, documentation and assignment of scores (or rating). Fact sheets of individual sub-indicators provide information on the maturity level, scope, description, objective, requirement, evidence to review, references, framework, rating scale and limitations and remarks. Particularly, the ‘evidence to review’ sections of the fact sheets enumerate specific items or provisions that should be specifically reviewed to assess and score each sub-indicator.

The GBT for CTO, in particular, has 30 sub-indicators, which disaggregate into 6 indicators ranging from CT01 (legal provisions, regulations and guidelines required to define regulatory framework of clinical trials oversight) to CT06 (mechanism in place to monitor regulatory performance and output). Like for other regulatory functions, the GBT assessment tool for CTO has sub-indicators that specifically measure and rate an NRA’s preparedness and capacity for managing public health emergencies.

The GBT also integrates the concept of ‘maturity level’ (adapted from ISO 9004:2018) [33] to assess and rate the performance level of the overall regulatory system and component regulatory functions using a scale ranging from 1 to 4. Level 1 represents the existence of some elements of regulatory system. Level 2 depicts evolving national regulatory system that partially performs essential regulatory functions. Level 3 describes a stable, well-functioning and integrated regulatory system (target of WHA resolution 67.20). Level 4 identifies regulatory system operating at advanced level of performance and continuous improvement. Be it through self- or formal-benchmarking, the GBT and its benchmarking methodology enables regulatory authorities and the WHO to identify strengths and areas of improvement, facilitate the formulation of an IDP to build upon strengths and address the identified gaps, prioritize interventions and monitor progress and achievements [3, 15]. With its application this way, the GBT serves as a useful tool that helps to classify regulatory systems and provides a structured approach to assess their configuration to achieve desired results, and if otherwise, defines the needed interventions to progress on system maturity.

### Description of GBT sub-indicators relevant to public health emergency preparedness for clinical trials oversight

This study included three of VaccTrain’s partner NRAs comprising Liberia Medicines and Health Products Authority (LMHRA - Liberia), Medicines Control Agency (MCA - The Gambia) and Pharmacy Board of Sierra Leone (PBSL - Sierra Leone). Data collected with the NRA worksheets as of November, 2021 (and validated by partner NRAs in confirmation of the latest status of required evidence) was used to assess the availability and implementation status of public health emergency–related sub-indicators as per specific indications in the WHO GBT. Specifically, we screened the WHO GBT for CTO for sub-indicators that made specific references to public health emergencies in their fact sheets and identified CT01.05, CT04.07 and CT06.04 sub-indicators. Briefly, while CT01.05 assesses legal provisions or regulations that allow NRAs to apply non-routine CT procedures such as fast-track or expedited decision-making of a Clinical Trial Application (CTA) during public health emergencies, CT04.07 probes procedures that are used to provide guidance for the review of CTAs in the event of emergencies. CT06.07 on the other hand, analyzes the internal tracking system for monitoring documented timelines of the CTA processing designed to be adaptable for routine and non-routine CTAs (e.g., health emergencies).

Based on our knowledge and experience, we included sub-indicators for NRA’s fundamental legal mandate to regulate CTs (i.e. CT01.01), and that for recognition and reliance (i.e. CT01.11) in the assessment. We however consciously avoided the *en masse* inclusion of all WHO “maturity level” (ML) 1 and 2 indicators as was done previously [4] simply on the technical grounds as highlighted above. Furthermore, we excluded all cross-cutting indicators, such as those that relate to quality management system, transparency, accountability, and communication and to the activities of the Ethics Committee. Lastly, we also omitted sub-indicators that aim to assess the established standard CTO framework for routinely screening, reviewing, authorizing and supervising CTs. Of the sub-indicators herein analyzed, three were ML1 & 2 legal provisions and regulations while the remaining two were ML3 sub-indicators that relate to the expected adjustments in an NRA’s approach to expediting the CT approval process during emergencies. The GBT CT sub-indicators herein assessed as relevant and directly related to regulatory preparedness for public health emergencies are summarized in Table 2. For a detailed background description of the individual sub-indicators here assessed, and the full list of indicators, sub-indicators and their fact sheets, please refer to the GBT’s CTO module [34].

**Table 2:** Global benchmarking tool sub-indicators for clinical trials oversight relevant to public health emergency preparedness

No.	(Sub-)Indicator	ML
	**CT01 Legal provisions, regulations and guidelines required to define regulatory framework of clinical trials oversight.**	
CT01.01	Legal provisions and regulations for clinical trials (CTs) oversight exist.	1
CT01.05	There are legal provisions or regulations covering circumstances in which the routine CT evaluation procedures may not be followed (e.g. for public-health interests)	2
CT01.11	Legal provisions / regulations allow NRAs to recognize and use relevant CT decisions, reports or information from other NRAs or from regional and international bodies.	1
	**CT04 Procedures established and implemented to perform clinical trials oversight.**	
CT04.07	The same policies are used for the evaluation of CT applications regardless of the applicant (e.g., domestic, foreign, public sector, or private sector)	3
	**CT06 Mechanism in place to monitor regulatory performance and output.**	
CT06.04	There are timelines for the assessment of CT applications and an internal tracking system to follow the targeted time frames	3

### Assessment of emergency-related sub-indicators for CTO

In assessing the emergency preparedness regulatory framework for CTO in our three partner countries, we devised two scoring and rating systems. The first was based on the scoring of individual “evidence to review” items from the fact sheets of each sub-indicator. Specifically, we scored each “evidence to review” item either 1 or 0 depending on the sufficiency of evidence provided for review (i.e., evidence provided = 1; no evidence provided = 0). Based on the tally of evidence provided scores for each sub-indicator, we then calculated the overall ‘percentage evidence available’ to demonstrate the real implementation status of that sub-indicator.

The second scoring system we employed was based on that developed and used by the WHO. Using this scoring system, we rated individual sub-indicators as (i) not implemented (NI), (ii) ongoing implementation (OI), (iii) partially implemented (PI), or (iv) fully implemented (I) based on the summary of evidence provided for “evidence to review” items from the fact sheets [15]. The ratings generally interpret as: (i) not implemented - no evidence of sub-indicator provisions established and implemented; (ii) ongoing implementation - evidence of recently drafted sub-indicator provisions exists but have not yet been followed; (iii) partially implemented - evidence of sub-indicator provisions exists but have only been followed for a limited period (usually < 2 years); (iv) fully implemented – evidence exist of sub-indicator provisions established and implemented [15].

### Mapping of training effectiveness to parameters that ensure successful implementation and achievement of outcomes

Data from the registry and results databases *ClinicalTrials.gov* and *Pactr.samrc.ac.za* were further used to aid in mapping country situational reports to the differential dynamics in CTs regulated by the individual NRAs.

## Supplementary Information

**Additional file 1.**

## Data Availability

The datasets generated and/or analysed during the current study are not publicly available due confidentiality and data protection rights but are available from the corresponding author on reasonable request.

## Abbreviations

GHPP: Global Health Protection Programme
CT: Clinical Trials
CTO: Clinical Trials Oversight
CTA: Clinical Trials Application
NRA: National Regulatory Authority
GBT: Global Benchmarking Tool
LMIC: Low- and Middle-Income Countries
ML: Maturity Level
SOP: Standard Operating Procedure
IDP: Institutional Development Plan
AVAREF: African Vaccine Regulatory Forum
AUDA-NEPAD: African Union Development Agency-New Partnership for Africa’s Development
