# Supplementary figures and images for: Leveraging WHO’s Global Benchmarking Tool to strengthen capacity in clinical trials oversight for public health emergencies: the GHPP VaccTrain model
Source: Global Health. 2022 Jun 20;18:63. doi: 10.1186/s12992-022-00854-0 (PMC9207864; doi:10.1186/s12992-022-00854-0)

A.

NRA 1

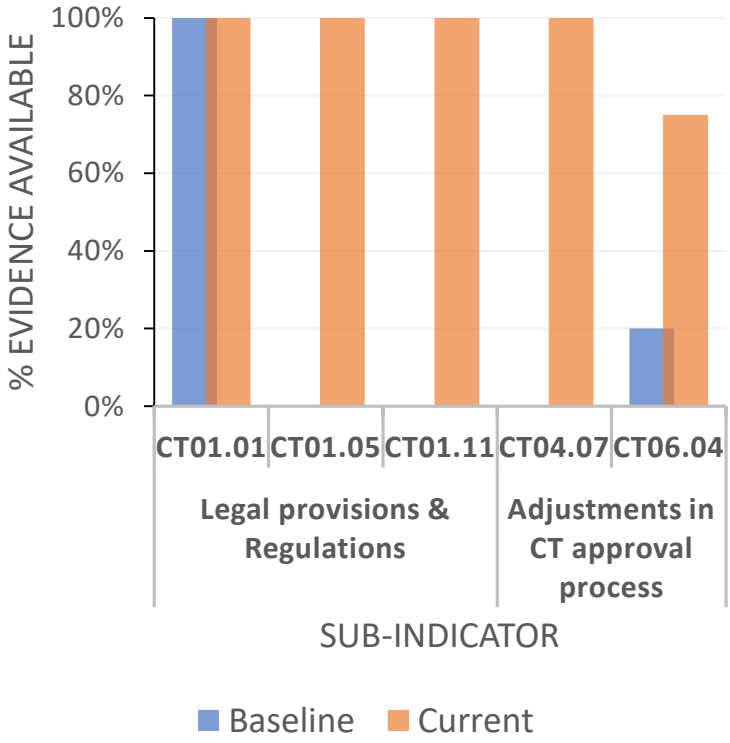

B.

NRA 2

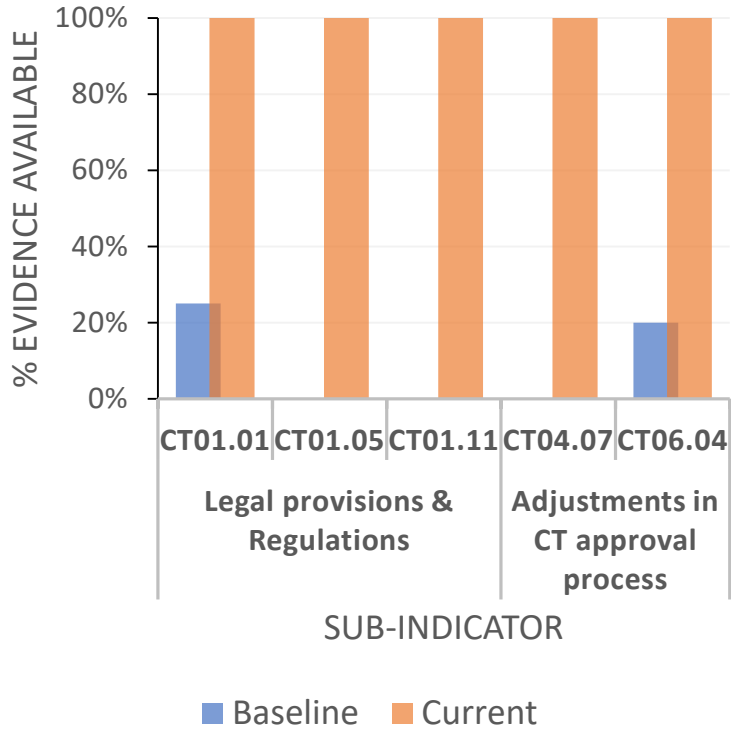

C.

NRA 3

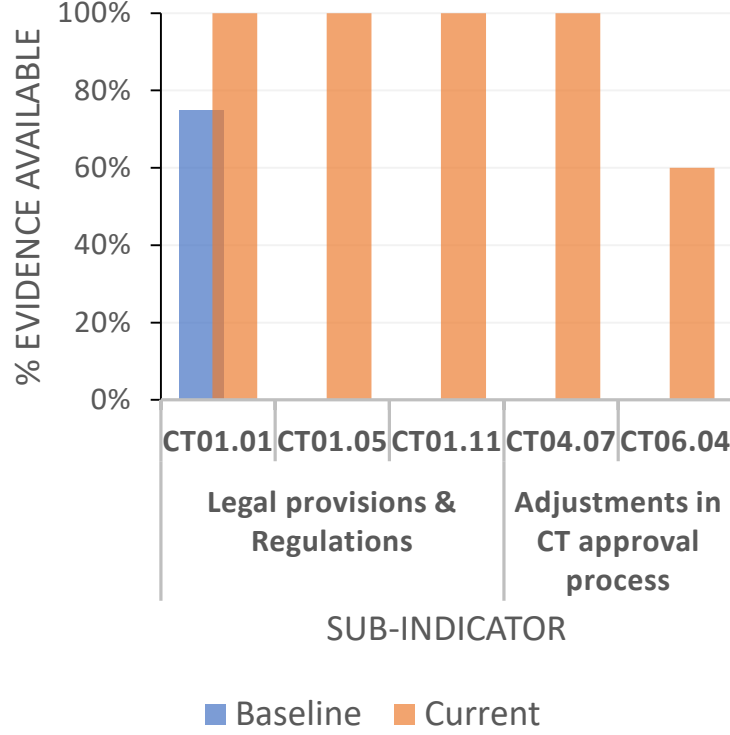

Supplement: Supplementary file 1 — Additional file 1. [file 12992_2022_854_MOESM1_ESM.pdf]
